# Supplementary material for: Impact of the COVID-19 pandemic on breast cancer screening indicators in a Spanish population-based program: a cohort study
Source: eLife. 2022 Jun 10;11:e77434. doi: 10.7554/eLife.77434 (PMC9212994; doi:10.7554/eLife.77434)
Supplement: Supplementary file 1. [file elife-77434-supp1.docx]

**Supplementary file 1**

**Crude logistic-regression models**

Appendix 1. Table 1. Multi-level logistic regression model for crude participation.

| **PARTICIPATION (POST-COVID-19)** | **OR** | **STD. ERROR** | **STATISTIC** | **P VALUE** | **95% CI (-)** | **95% CI (+)** |
| --- | --- | --- | --- | --- | --- | --- |
| FIRST TIME INVETEES | 0.863 | 0.0278 | -4.58 | 4.71e- 6 | 0.81 | 0.919 |
| PREVIOUS NON-PARTICIPANTS | 1.09 | 0.0452 | 1.99 | 4.64e- 2 | 1 | 1.18 |
| REGULAR PARTICIPANTS | 0.663 | 0.019 | -14.4 | 7.34E-47 | 0.627 | 0.701 |
| IRREGULAR PARTICIPANTS | 0.957 | 0.0482 | -0.865 | 3.87e- 1 | 0.868 | 1.06 |

Appendix 1. Table 2. Multi-level logistic regression model for crude recall.

| **RECALL (POST-COVID-19)** | **OR** | **STD. ERROR** | **STATISTIC** | **P VALUE** | **95% CI (-)** | **95% CI (+)** |
| --- | --- | --- | --- | --- | --- | --- |
| PREVALENT SCREENING | 0.736 | 0.101 | -2.23 | 2.59e- 2 | 0.562 | 0.964 |
| INCIDENT SCREENING | 0.829 | 0.0649 | -2.39 | 1.67e- 2 | 0.711 | 0.967 |

Appendix 1. Table 3. Multi-level logistic regression model for crude false positives.

| **FALSE POSITIVES (POST-COVID-19)** | **OR** | **STD. ERROR** | **STATISTIC** | **P VALUE** | **95% CI (-)** | **95% CI (+)** |
| --- | --- | --- | --- | --- | --- | --- |
| PREVALENT SCREENING | 0.939 | 0.15 | -0.392 | 6.95e- 1 | 0.686 | 1.29 |
| INCIDENT SCREENING | 0.708 | 0.0665 | -3.68 | 2.38e- 4 | 0.589 | 0.851 |

Appendix 1. Table 4. Independent logistic regression for crude cancer detection.

| CANCER DETECTION (POST-COVID-19) | Estimate | Std. Error | z value | Pr(>\|z\|) | ODDS | OR post/pre | IC(95%) inf. | IC(95%) sup. |
| --- | --- | --- | --- | --- | --- | --- | --- | --- |
| PREVALENT SCREENING | -0.08 | 0.28 | -0.29 | 0.78 | 0.92 | 0.92 | 0.52 | 1.56 |
| INCIDENT SCREENING | -0.24 | 0.18 | -1.32 | 0.19 | 0.78 | 0.89 | 0.66 | 1.19 |
